# COVID-19, MERS and SARS with Concomitant Liver Injury—Systematic Review of the Existing Literature

**DOI:** 10.3390/jcm9051420

**Published:** 2020-05-11

**Authors:** Michał Kukla, Karolina Skonieczna-Żydecka, Katarzyna Kotfis, Dominika Maciejewska, Igor Łoniewski, Luis. F. Lara, Monika Pazgan-Simon, Ewa Stachowska, Mariusz Kaczmarczyk, Anastasios Koulaouzidis, Wojciech Marlicz

**Affiliations:** 1 Department of Internal Medicine and Geriatrics, Jagiellonian University Medical College, 2 Jakubowskiego St., 30-688 Cracow, Poland; kuklamich@poczta.onet.pl; 2 Department of Endoscopy, University Hospital in Cracow, 2 Jakubowskiego St., 30-688 Cracow, Poland; 3 1st Infectious Diseases Ward, Gromkowski Regional Specialist Hospital, Wroclaw, 5 Koszarowa St., 50-149 Wroclaw, Poland; monika.pazgan.simon@gmail.com; 4 Department of Human Nutrition and Metabolomics, Pomeranian Medical University in Szczecin, 71-460 Szczecin, Poland; karzyd@pum.edu.pl (K.S.-Ż.); dmaciejewska.pum@gmail.com (D.M.); sanprobi@sanprobi.pl (I.Ł.); ewa.stachowska@pum.edu.pl (E.S.); 5 Department of Anaesthesiology, Intensive Therapy and Acute Intoxications, Pomeranian Medical University in Szczecin, 70-111 Szczecin, Poland; katarzyna.kotfis@pum.edu.pl; 6 Division of Gastroenterology, Hepatology, and Nutrition, The Ohio State University Wexner Medical Center, Columbus, OH 43210, USA; Luis.Lara@osumc.edu; 7 Department of Infectious Diseases, Wroclaw Medical University, 5 Koszarowa St., 50-149 Wroclaw, Poland; 8 Department of Clinical and Molecular Biochemistry, Pomeranian Medical University in Szczecin, 70-111 Szczecin, Poland; mariush@pum.edu.pl; 9 Centre for Liver & Digestive Disorders, Royal Infirmary of Edinburgh, Edinburgh EH16 4SA, UK; akoulaouzidis@hotmail.com; 10 Department of Gastroenterology, Pomeranian Medical University, 71-252 Szczecin, Poland

**Keywords:** SARS-CoV-2, coronavirus, liver, pandemic, SARS, MERS, COVID-19

## Abstract

The novel coronavirus SARS-CoV-2 (severe acute respiratory syndrome coronavirus 2) infection has been predominantly linked to respiratory distress syndrome, but gastrointestinal symptoms and hepatic injury have also been reported. The mechanism of liver injury is poorly understood and may result as a consequence of viral hepatitis, systemic inflammatory response, gut barrier and microbiome alterations, intensive care treatment or drug toxicity. The incidence of hepatopathy among patients with coronavirus disease 2019 (COVID-19) is unclear, but studies have reported liver injury in patients with SARS and Middle East respiratory syndrome (MERS). We aimed to systematically review data on the prevalence of hepatic impairments and their clinical course in SARS and MERS *Coronaviridae* infections. A systematic literature search (PubMed/Embase/Cinahl/Web of Science) according to preferred reporting items for systematic review and meta-analysis protocols (PRISMA) was conducted from database inception until 17/03/2020 for studies that evaluated the incidence of hepatic abnormalities in SARS CoV-1, SARS CoV-2 and MERS infected patients with reported liver-related parameters. A total of forty-three studies were included. Liver anomalies were predominantly mild to moderately elevated transaminases, hypoalbuminemia and prolongation of prothrombin time. Histopathology varied between non-specific inflammation, mild steatosis, congestion and massive necrosis. More studies to elucidate the mechanism and importance of liver injury on the clinical course and prognosis in patients with novel SARS-CoV-2 infection are warranted.

## 1. Introduction

The novel coronavirus named SARS-CoV-2 (severe acute respiratory syndrome coronavirus 2) was declared a public health emergency of international concern on 30 January, 2020 after the outbreak of severe pulmonary infections of unknown cause that started in Wuhan, Hubei province, China, in December 2019 [1,2]. The term coronavirus disease 2019 (COVID-19) was designated by the World Health Organization (WHO) on the 11th of February 2020 to be used for the severe pneumonia associated with SARS-CoV-2 infection. On Wednesday, 11 March 2020, COVID-19 was declared a pandemic by the WHO, as it affected more countries and threatened the lives of patients worldwide [3]. Several groups of investigators have already reported frequent gastrointestinal symptoms in COVID-19 patients which may be associated with increased disease severity [4,5], including prolonged coagulation times and elevated liver serum tests compared to those without digestive symptoms [6,7]. Hepatopathy has been associated with disease severity with two other highly pathogenic coronavirus strains—severe acute respiratory syndrome coronavirus 1 (SARS-CoV-1) and the Middle East respiratory syndrome coronavirus (MERS-CoV) [8]. Recent clinical studies of COVID-19 indicate that hepatic injury presents with elevated transaminases, elevated bilirubin, prolonged prothrombin time and hypoproteinemia [9] and the severity of blood test anomalies may predict a poorer outcome. Bangash et al. reported that mild laboratory anomalies were not prognostic of outcomes [10]; however, 58% to 78% of patients with severe COVID-19 associated disease presented with varying degrees of liver injury [11], therefore clinically significant liver dysfunction may be an important feature of and may predict the prognosis of COVID-19 [2,9,12]. The pathogenesis is likely multifactorial including: (i) viral hepatitis (hepatic viral replication); (ii) hypoxic hepatitis (secondary to respiratory failure); (iii) hepatic congestion associated with mechanical ventilation (high levels of positive end-expiratory pressure (PEEP)); (iv) drug toxicity (antiviral medications, anti-malaria medications, antibiotics, steroids); (v) immune response (virally induced intrahepatic cytotoxic T cells and Kupffer cells) and (vi) gut vascular barrier and microbiota alterations [10]. In the most severe cases of COVID-19 hepatic dysfunction has been associated with an activation of coagulation and fibrinolysis accompanied by thrombocytopenia [12].

Previous studies regarding SARS and MERS indicated that approximately 60% of patients presented with hepatopathy, mostly in the form of elevated liver enzymes [13]. Severe COVID-19 disease has been associated with a high prevalence of abnormal aminotransferase levels; however, they may also be of non-hepatic origin [12]. We aimed to systematically review the existing data to determine the correlation of hepatic involvement in patients with either SARS-CoV-1, MERS-CoV or SARS-CoV-2 infection, and determine a relationship with outcomes, especially prognosis in patients with COVID-19.

## 2. Materials and Methods

### 2.1. Search Strategy and Selection Criteria

The preferred reporting items for systematic review and meta-analysis protocols (PRISMA) methodology [14] was used as the search strategy. Two blinded authors (K.S.-Ż. and D.M.) performed a systematic search in the following databases: PubMed/Medline/Embase/Cinahl/Web of Science from database inception until 17 March 2020. The search string utilized was: ((“coronaviridae” (Terms) OR “coronaviridae” (All Fields)) OR (“coronavirus infections” (MeSH Terms) OR (“coronavirus” (All Fields) AND “infections”(All Fields)) OR “coronavirus infections” (All Fields) OR (“coronavirus” (All Fields) AND “infection” (All Fields)) OR “coronavirus infection” (All Fields))) AND (“liver” (MeSH Terms) OR “liver” (All Fields)) AND (“humans” (MeSH Terms) OR “humans” (All Fields) OR “human” (All Fields)). We did not include reviews, meta-analyses and systematic reviews; however, any commentary, editorial, letter to editors, abstract where clinical data were provided was included for further review and data extraction. After screening of the aforementioned databases, the search was supplemented by manual review of references covering the topic of interest.

### 2.2. Inclusion Criteria

- Human retrospective studies, including case reports and case series reporting clinical liver-related data in patients infected with SARC-CoV-1, SARS-CoV-2 and MERS;
- Any of the following clinical data: alanine aminotransferase (ALT)/aspartate aminotransferase (AST)/gamma-glutamyl-transpeptidase (GGTP)/alkaline phosphatase (ALP)/lactate dehydrogenase (LDH)/creatinine (Cr)/bilirubin (BIL)/total protein (TP)/albumin (ALB)/international normalised ratio (INR)/prothrombin time expressed either qualitatively (percent of abnormal results) or quantitatively (PT);
- Post-mortem studies reporting liver histopathology.

Pediatric patients were excluded; however, when a study reported data on both children and adults the data were evaluated separately for persons above the age of 18 years. In addition, for studies originating in China we extracted data only for abstracts in English.

### 2.3. Data Extraction and Analysis

At least two authors (K.S.-Ż., D.M. and M.K. (Michał Kukla)) independently abstracted data on basic study characteristics (type of coronavirus, study aim, type of study, i.e., in vivo/post mortem), study subjects (age, comorbidities), intervention (drugs, intensive care treatment) and liver related parameters. When abstracting data from figures, WebPlot digitizer software was used (https://automeris.io/WebPlotDigitizer/). The data were stored in an Excel file by each investigator and after the comparison of the file content all discrepancies were solved by the third author by consensus. The significance of the analyzed studies was arbitrarily assigned as follows: strong—full text studies with data on more than 30 patients, middle—studies comprising a small number of patients (<30), weak—case reports and abstracts with no access to full text.

### 2.4. Risk of Bias Assessment

Two authors (K.S.-Ż. and D.M.) independently assessed the risk of bias using the Strengthening the Reporting of OBservational Studies in Epidemiology (STROBE) assessment [15]. However, item 16 (unadjusted estimates, confounder-adjusted estimates, category boundaries, translating estimates of relative risk into absolute risk for a meaningful period), was omitted as it was not applicable to the present study. Each item was evaluated, and 1 point was added for each when fulfilled. Therefore, the rating was the sum of points received for each STROBE item. The highest number of points obtainable in each study was 31.

## 3. Results

### 3.1. Descriptive Data

**Figure 1:**
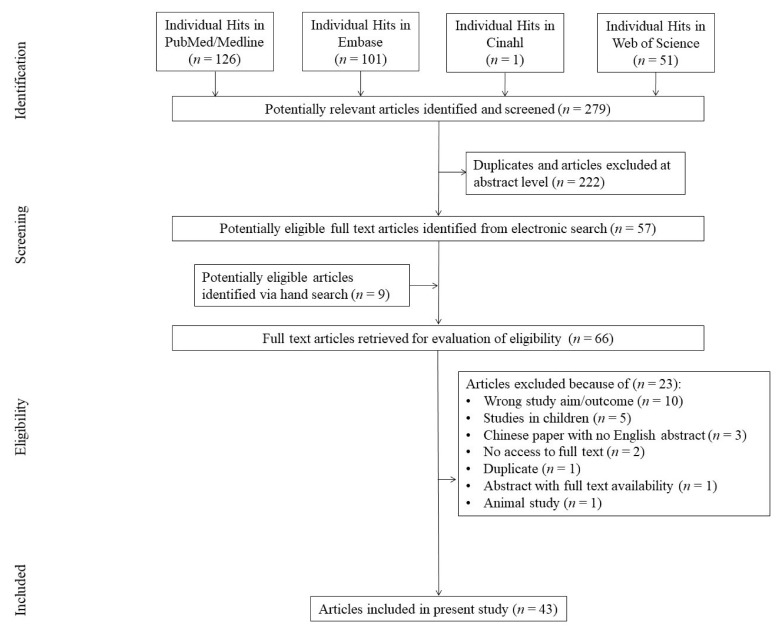
Study and sample characteristics.

The initial search yielded 279 results, of which 222 were excluded after reviewing titles and abstracts. Fifty-seven manuscripts were fully reviewed, plus 9 more studies which were manually found. Subsequently 23 studies were excluded due to lack of liver-related clinical data (*n* = 10); pediatric patients (*n* = 5); no access to English abstract (*n* = 3); no access to full texts (*n* = 2); and one each (*n* = 1) including animal study, duplicate and incomplete abstract. Thus, 43 articles comprised the study group [2,4,8,12,13,16,17,18,19,20,21,22,23,24,25,26,27,28,29,30,31,32,33,34,35,36,37,38,39,40,41,42,43,44,45,46,47,48,49,50,51,52,53] (Figure 1).

Most of the studies (*n* = 35) [2,4,12,13,16,17,18,19,20,21,22,23,24,25,27,30,31,32,33,35,36,37,38,40,41,42,44,45,46,47,48,49,50,51,52] were observational and retrospective. We extracted data from eleven (*n* = 11) English abstracts provided for Chinese articles [17,26,30,32,34,35,37,38,42,44,48] and for one (*n* = 1) commentary [19].

There were eight (*n* = 8) post-mortem studies (7 full texts, 1 abstract) [8,26,28,29,34,39,43,53]. There were 11 (*n* = 11) [2,4,12,16,17,18,19,20,21,22,23] studies focusing on SARS CoV-2 and 23 (*n* = 23) [13,24,25,26,27,28,29,30,31,32,33,34,35,36,37,38,39,40,41,42,43,44,45] which reported data on patients infected with SARS CoV-1. MERS had the last amount of data (*n* = 9) [8,46,47,48,49,50,51,52,53]. Overall, we analyzed clinical characteristics of 4591 subjects, most with SARS CoV-2 (*n* = 2541), followed by SARS CoV-1 (*n* = 1894) and MERS (*n* = 156). The mean/median age, when provided, was between 33 and 45.21 years. Comorbidities were reported in 14 studies including hypertension (HTN) in 306, diabetes mellitus (DM) in 171 and cardiovascular diseases (CVD) in 112 patients. Obesity status (*n* = 7) and body mass index (BMI) value (30.5 kg/m^2^) were provided in a single study each. Other comorbidities included asthma, chronic obstructive pulmonary disease (COPD), kidney diseases and others, with a total number of 398 cases. Treatment data were provided in 24 studies; however, drugs dosages and duration of intervention were predominantly missing. Mechanical ventilation was reported in 407 patients in 24 studies. The reported study, patient and treatment characteristics for all three types of coronaviruses are provided in Table 1.

### 3.2. Liver-Related Outcomes

The most frequently reported data were concentrations of ALT (*n* = 19) and AST (*n* = 16), followed by bilirubin (*n* = 14) and serum creatinine (*n* = 12). Raw data on albumin content, prothrombin time, LDH and ALP were reported by 10, 8, 6 and 4 studies, respectively. Quantitative data including number and percentage of patients with abnormal tests were extracted. The most commonly reported were ALT (*n* = 14), AST (*n* = 9) and creatinine (*n* = 6). Normal ranges varied by study (ALT: >40–53 U/L; BIL: >17–21 µmol/L; LDH: >245–250 U/L; ALB: <40–42 g/L; PT: >12.5–16 s; CRL: >111–133 µmol/L). The data are presented in Table 2. Twelve studies reported liver histopathology, seven (*n* = 7) were autopsy studies. The details are provided in Table 3.

### 3.3. Risk of Bias (ROB)

The ROB evaluation was performed using the STROBE assessment tool in 28 full text manuscripts. None of the studies reached 31 points. None of the studies described methods to address risk of bias within their observations. The biggest variations occurred regarding the study size and statistical methods, in particular, the quantitative variables data. The mean number of points achieved was 26.54 ± 2.5 points. The lowest number of points achieved was 23, and the highest was 30. The detailed scoring is reported in Supplementary Table S1.

## 4. Discussion

A number of studies have documented SARS infection associated with liver injury as manifested by mild and moderate elevation of ALT and/or AST at the onset of the disease [11,19,54]. Angiotensin-converting enzyme 2 (ACE2) has been established as a membrane receptor for SARS-CoV cell entry [55]. ACE2 was shown to be abundantly expressed in several cell types, including not only lung type II alveolar cells (AT2) but also monocytes and vascular endothelial cells [56]. Recently, Meng-Yuan et al. compared ACE2 expression levels across 31 human tissues using the datasets from the Genotype-Tissue Expression (GTEx) project and the Cancer Genome Atlas (TCGA) program. The researchers were able to report highest ACE2 expression in the small intestine, testis, kidneys, heart, thyroid and adipose tissue and medium expression in the lungs, colon, liver, bladder and adrenal gland [57]. The study by Paizis et al. [58] using immunohistochemistry confirmed ACE2 expression in hepatocytes of healthy human liver which increased in chronic liver diseases and during hypoxia. Therefore, gastrointestinal manifestations of the disease are likely a result of alterations of the gut-liver and gut-pulmonary axes. As documented by Spadoni et al. [59] the gut vascular barrier (GVB) is vulnerable to noxious infectious agents with the end result of systemic dissemination of pathogens. In patients with coeliac disease (CD) with elevated serum transaminases GVB alterations may be responsible for liver damage [59]. Although gut barrier disruption has not been reported to be a pre requisite for nonalcoholic steatohepatitis (NASH) development and belongs to the ‘multiple hit’ pathogenesis of disease progression [60], more recent observations in preclinical models have shown that disruption of epithelial and vascular barriers in the intestine were early events reported in NASH [61]. Gut-liver axis deterioration has also been described in patients with chronic liver diseases who develop sepsis and multiorgan failure [62,63]. Zhou et al. in their recent retrospective cohort study of 191 patients reported that more than half of hospitalized patients developed sepsis as a common complication of SARS-CoV-2 infection [18]. However, data on the pathogenesis of sepsis in COVID-19 patients and the potential role of gut-liver axis and hepatic dysfunction remain unknown.

### 4.1. COVID-19 Patients and Liver Injury

In our current study we evaluated the data on laboratory and liver pathology parameters of 4591 persons, infected with SARS CoV-2 (*n* = 2541), followed by SARS CoV-1 (*n* = 1894) and MERS (*n* = 156). All the studies analyzed were retrospective and/or case reports. The mean/median age of all study patients ranged between 33 and 45.21 years. Main comorbidities reported in 14 studies included hypertension (HTN) in 306, diabetes mellitus (DM) in 171 and cardiovascular disease (CVD) in 112 patients. Jin et al. in their retrospective epidemiological study evaluating clinical and virologic characteristics of 74 COVID-19 patients manifesting with gastrointestinal symptoms (nausea, vomiting, diarrhea) reported increased AST, but not ALT levels, which were significantly higher in patients with COVID-19 with gastrointestinal (GI) symptoms than in those without GI symptoms (29.35 vs. 24.4, *p* = 0.02). Gastrointestinal symptoms were associated with more severe course of infection (in 23% of patients compared to 8% of patients without alimentary tract symptoms). Additionally, patients with gastrointestinal tract involvement had significantly higher rates of fever >38.5 °C, fatigue, shortness of breath and headache. Based on initial univariate analysis of epidemiological, clinical and laboratory variables the authors identified 11 significant risk factors for severe/critical COVID-19, including increased operating rooms (ORs) of age ≥50 years, period between illness onset and hospital visit, sputum production, any existing medical condition, multiple lung infection, ALT and lactate dehydrogenase (LDH) levels, glucose and C reactive protein (CRP) concentrations, as well as decreased OR of the infected area [4]. Based on these variables Jin, et al. concluded that sputum production and increased LDH/glucose levels were independent risk factors for severe/critical COVID-19 in patients with GI symptoms [4].

Most of the patients included in our systematic review were younger, below the age of 50. Generally only ALT/AST levels were frequently reported, with scarce information on LDH, previous infections, glucose or CRP. These data were also missing in a recent meta-analysis by Parohan et al., who concluded that the incidence of liver injury may be higher in patients with severe COVID-19 infection [64]. Similarly Xu et al. [11] summarized the reports of liver injury caused by SARS-CoV, MERS-CoV and SARS-CoV-but were unable to define the mechanisms of liver injury associated with SARS-CoV-2 infection. In our systematic review the risk of severe illness could not be predicted using the variable which were reported.

### 4.2. Hepatic Dysfunction Associated with ICU Procedures

The need for organ support and intensive care treatment, regardless of the reason (i.e., acute hypoxemic respiratory failure, shock, heart failure) during the COVID-19 outbreak has proven to be substantial resulting in significant strain on health care systems around the world. Based on the available data, standard of care was observed for ICU treatment and procedures for patients with SARS-CoV-2 [2,12,18,65] For sepsis diagnosis and treatment, the 2016 Third International Consensus Definition for Sepsis and Septic Shock [66] guidelines were followed; acute respiratory distress syndrome (ARDS) was diagnosed according to the Berlin Definition [67], and acute kidney injury was diagnosed according to the Kidney Disease Improving Global Outcomes (KDIGO) clinical practice guidelines [68].

Following recent guidelines, early endotracheal intubation to decrease the risk of virus aerosolization was recommended in patients with acute hypoxemic respiratory failure, including high positive end-expiratory pressure (PEEP) and prone ventilation for 12 to 16 h per day [3,69]. Hepatic dysfunction has been associated with mechanical ventilation, namely high levels of PEEP that contributes to hepatic congestion. High levels of PEEP (18–20 cm H_2_O), as recommended for treatment of respiratory failure associated with COVID-19 [3], may significantly increase right atrial pressure, leading to obstructed venous return from the inferior vena cava and venous congestion. However, according to the available data, liver blood test abnormalities were also found in patients with COVID-19 without mechanical ventilation. Based on our systematic search the data on the effect or association of mechanical ventilation and levels of PEEP and liver test anomalies are inconclusive.

As expected, non-survivors presented with higher sequential organ failure assessment (SOFA), quick sequential organ failure assessment (qSOFA) and pneumonia CURB-65 (confusion, urea, respiratory rate, blood pressure, age above 65) scores, which indicates higher disease severity and multi-organ involvement [18]. The frequency of complications was higher in non-survivors than survivors, with sepsis and ARDS being the most frequently observed complication, followed by heart failure and septic shock [12,18]. The mortality rates among patients requiring ICU admission ranged from 16% to 78% [12,18,65,70].

Respiratory support requirement was substantial. In studies regarding the ICU population, the reported use of non-invasive ventilation ranged from 11% [65] to 62% [70] and this value included patients receiving high-flow nasal cannula [2]. A retrospective case series by Grasselli et al. [65] involving critically ill patients with laboratory-confirmed COVID-19 admitted to ICUs in Lombardi, Italy found that 99% of patients required respiratory support, including endotracheal intubation and invasive ventilation in 88%. The need for invasive mechanical ventilation in this patient population was higher than that recently reported for other ICU’s ranging from 30% to 71% [12,18,20,70,71]. ExtraCorporeal Membrane Oxygenation (ECMO) therapy was used in only 0.5%–2% of cases [2,12,18]. None of the studies reported hepatic failure as the main cause of ICU admission.

### 4.3. Hepatotoxicity Related to COVID-19

Studies in SARS showed that the genetic material of the SARS-CoV-2 was present in the liver tissue, confirming direct hepatocyte infection and dysfunction. No fibrosis or fibrin deposition was found [13]. Hepatotoxicity associated with SARS-CoV-2 or any other coronavirus could therefore be a result of direct viral hepatitis or secondary to drug toxicity from antiviral drugs, steroids and/or antibiotics. The effect of an enhanced inflammatory response associated with COVID-19 cannot be ruled out. Studies reporting RNA sequencing data from intracellular viral load of two independent patient cohorts showed a significant increase of ACE2 expression in cholangiocytes (59.7%) as compared with hepatocytes (2.6%). This suggests an affinity of the 2019-nCoV to the bile ducts [72]. In our analysis only 12 studies reported liver histopathology, but none in SARS-CoV-2 infected patients. The descriptions included: (i) non-specific inflammation in postmortem biopsy; (ii) congestion, hemorrhage and focal perivenular loss of hepatocytes (iii) macrovesicular perivenular steatotic change, (iv) sinusoidal congestion and (v) hemorrhage or (vi) focal perivenular loss of hepatocytes. Massive hepatic necrosis was reported in only one case. Few of the studies explored the viral load in hepatic tissue, which may be relevant in light of data reporting the novel m6 A methylation loci in the S protein of SARS-CoV-2 as an underlining mechanism for its virulence and transmission capacity [4]. A recent post-mortem case study published during revisions of this review reported moderate microvesicular steatosis and mild lobular and portal activity in a patient who expired due to COVID-19, suggesting it was caused by either SARS-CoV-2 infection, but drug-induced liver injury could not be ruled out [73]. Further research regarding SARS-CoV-2 hepatotoxicity with potential new therapeutic options is necessary [7].

### 4.4. Comorbidities and Liver Injury in COVID-19 Patients

The effect of having underlying chronic liver disease remains unclear as there were a small number of subjects with chronic hepatitis B and liver cirrhosis. The association between pre-existing liver disease and SARS-CoV-2 infection needs further investigation.

Patients with type 2 diabetes mellitus (T2DM) and hypertension are not only more vulnerable to COVID-19 but also have a more serious disease course. The study by Guan et al. [2] found hypertension in 15%, T2DM in 7.4% and chronic hepatitis B in 2% of patients with COVID-19. Subjects with T2DM and/or obesity which together with arterial hypertension are components of metabolic syndrome would be expected to have a higher incidence of nonalcoholic fatty liver disease (NAFLD) or nonalcoholic steatohepatitis (NASH) and be at higher risk for hepatic manifestations of COVID-19, but there was insufficient data to determine an association. Another study by Wang et al. [12] showed hypertension in 31%, CVD in 14%, T2DM in 14% and preexisting chronic liver disease in 3%. Patients with severe COVID-19 requiring ICU care were twice as likely to have these co-morbidities. There was no particular data on NAFLD in this study. The average aminotransferases levels were within normal range for the extended cohort of patients with COVID-19, but a sub-analysis of patients with mild and severe disease revealed significantly elevated ALT and AST levels in those requiring ICU care. Total serum bilirubin concentration was also higher in these patients. The association of metabolic anomalies and higher AST and ALT levels in patients with severe course of disease suggests increased susceptibility to the hepatotoxic effect of the virus. The study by Zhou et al. [18] also described a two-fold increase of T2DM (19% of the cohort), hypertension (30%) and CVD (15%) in patients with a fatal outcome. Data on preexisting liver disease and metabolic abnormalities was insufficient for study. Serum albumin concentration was significantly lower in non-survivors. In addition, ferritin and interleukin 6 (IL-6) levels alongside ALT activity increased significantly in patients with more severe disease. Both ferritin and IL-6 have been described with non-favorable course of liver injury in general [18]. Contemporaneous increase in ferritin, IL-6 and ALT levels associated with decreased albumin concentration suggest more significant liver involvement in the course of COVID-19.

Another study, by Yang et al. [20] compared 52 patients with severe COVID-19. There was higher incidence of T2DM in non-survivors. Liver dysfunction occurred with similar frequency when comparing survivors with non-survivors (30% vs. 28%). However, in non-survivors, higher serum bilirubin, lower platelet count and prolonged prothrombin time were found. In the study by Shi et al. [23] 81 patients, all with pneumonia, including 7 patients with liver cirrhosis, were analyzed in respect to the severity of radiological findings on computed tomography (CT) scan. Patients with subclinical disease had significantly lower AST but not ALT activity compared to those with overt pulmonary symptoms. Liver tests including serum albumin, thrombin time, fibrinogen, platelet count and bilirubin were not different between those with and without cirrhosis, but larger studies are necessary to understand the effect of cirrhosis on COVID-19 infection and outcomes. The study by Jin et al. [4] compared patients infected with SARS-CoV-2 with and without gastrointestinal (GI) symptoms (nausea, vomiting, diarrhea). The study showed that patients with GI symptoms were more likely to experience a severe/critical disease course and had higher rates of >38.5 °C-temperature and shortness of breath. More patients with GI symptoms had preexisting chronic liver disease (11% vs. 3%). Additionally, when analyzed separately, patients with chronic liver disease also had a statistically insignificant increased risk for severe/critical disease (23% vs. 8%). Patients with GI symptoms had lower levels of albumin and upregulated AST activity and CRP concentration and depletion of lymphocyte count. There was no difference in ALT activity, prothrombin time and bilirubin levels when compared to patients without GI disease. These results suggest an association between the presence of GI symptoms alongside with preexisting chronic liver disease and a more severe form of COVID-19. Importantly, increased ALT activity was found to be an important identifier of severe/critical COVID-19. Additionally, patients with GI symptoms were more prone to respiratory complications and liver injury (6.8% vs. 2.1%; 17.6% vs. 2.1%, respectively). These results show that in some patients SARS-CoV-2 exerts higher affinity to the gastrointestinal tract including the liver with an increased risk for hepatic injury. Similar to SARS-CoV, SARS-CoV-2 enters the cell using ACE2 receptors [72,74] which are expressed in hepatocytes and bile duct epithelial cells (cholangiocytes). The ACE2 has also been found to be expressed on cholangiocytes [75]. Cholangiocytes play an essential role in liver regeneration and immune response [76] This suggests that the liver injury in patients with COVID-19 may result from cholangiocyte damage, and not through hepatocytes. As mentioned above, IL-6 levels were significantly upregulated especially in more severe and critical patients [18]. Normally, IL-6 has an hepatoprotective effect and adjusts hepatic regeneration. Increased levels of IL-6 derange liver regeneration, stimulate hepatocytes to produce various proinflammatory cytokines and potentiates necroinflammatory activity and liver injury [77,78] Postmortem biopsies of patients with COVID-19 disease showed moderate microvascular steatosis and mild lobular and portal inflammatory activity only. It is unclear if this is directly due to infection or a result from drug-induced liver injury.

Obesity was reported to be an additional factor of severe COVID-19 illness [79]. However, obesity status was evaluated in only two studies in our systematic search reporting only seven patients (*n* = 7) and one case report. Other reported comorbidities included asthma, COPD, kidney diseases and others with a total number of 398 patients. A total of 407 patients required mechanical ventilation; however, data were scarce and reported only in 19 studies, which limits the conclusions on potential mechanisms and role of other factors associated with liver failure in COVID-19 patients.

### 4.5. SARS-CoV-1 and Liver Injury

SARS-CoV-1 was also suggested to be associated with liver injury. Autopsies of SARS patients found large numbers of virus particles not only in the lungs but also in the hepatocytes and hepatic vascular endothelial cells [80,81]. The retrospective study by Chan et al. [24] investigated the pattern of hepatic damage as well as the effect of chronic hepatitis B on the clinical outcome of SARS. The study included 12 HBsAg-positive (including two with cirrhosis) and 106 HBsAg-negative SARS patients. Among ten non-cirrhotic HBsAg-positive patients 3 were admitted for chronic hepatitis B flare-up, 5 had elevated and 2 had normal ALT levels. Among the 106 HBsAg-negative patients, 16 (15%) patients had elevated ALT activity on admission. None of these patients had comorbid illnesses or had taken any medications. However, 71 patients with normal ALT on admission experienced an increase during hospitalization. ALT activity was not associated with severity of SARS infection and eventual complications. Overall, 66 of 80 (83%) patients who experienced ALT elevation had normalization of ALT levels on discharge and another 10 (13%) patients had decreasing levels on follow-up. Histopathology of autopsy specimens showed a lack of acute hepatitis changes or hepatic necrosis. Coronavirus particles were not detected by electron microscopy. The clinical outcomes of chronic hepatitis B patients were not different from those of HBsAg-negative patients. The absence of specific hepatic lesions in hepatitis B virus and SARS-CoV-1 coinfected patients on autopsy suggests no correlation between SARS-CoV-1 and chronic hepatitis B infection. Also, the presence of hepatitis B virus infection did not influence the clinical outcomes of SARS. The authors concluded that the liver derangement in SARS was likely a nonspecific transient reaction caused by the infection rather than by a direct cytopathic effect of SARS-CoV-1 Another study by Chan et al. [25] encompassed 294 patients, including 30 with chronic hepatitis B. Elevated ALT activity on admission was found in 24% patients, while 69% of patients developed elevated ALT during the course of illness. Twenty-eight (9.5%) patients had ALT over 5 × upper limit of normal (ULN), 7 patients had elevated serum bilirubin and 10 had abnormal prothrombin time. Forty (29%) patients had experienced elevated ALP during the course of illness. None of the patients developed hepatic encephalopathy. Seven of the 28 (25%) patients died of SARS and multi-organ failure. On the other hand, 5 of 89 (6%) patients who had persistently normal ALT died. Among the 180 patients who had elevated ALT levels, 128 (71%) subsequently normalized and 37 (21%) patients had improved ALT level on follow-up visit. There were 28 patients who had peak ALT over 5 × ULN with significant male predominance and co-morbidities. A cut-off ALT >5 × ULN was associated with oxygen desaturation OR 3.24 (95% CI 1.23–8.59, *p* = 0.018), ICU care OR 3.70 (95% CI 1.38–9.89, *p* = 0.009), mechanical ventilation OR 6.64 (95% CI 2.22–19.81, *p* = 0.001) and death OR 7.34 (95% CI 2.28–24.89, *p* = 0.001). Co-existing chronic hepatitis B was not associated with higher ALT level, increased risk of oxygen desaturation, ICU admission, mechanical ventilation or mortality. The authors concluded that reactive hepatitis with transiently upregulated transaminases is a common complication of SARS-CoV infection. However, contrary to the previous study, severe hepatitis indicated by higher ALT activity, had a negative impact on outcomes of SARS. The study by Yang et al. [39] with 168 patients with SARS included 17 who were HBsAg or HBeAg-positive. The authors measured variables at four time points during hospitalization (day of admission, 1st, 2nd and 3rd week after admission). Abnormal ALT activity was observed in 52.5%, 71.8%, 85.7% and 85.2% of patients, respectively, with the highest average ALT in the 4th week of hospitalization (56.07, 86.46, 106.69 and 111.32 U/L, respectively). The average levels of serum albumin decreased during the hospitalization (37.25, 35.82, 34.49 and 34.26 g/L, respectively). AST activity was slightly increased (1.5 × ULN), while total bilirubin concentration remained within normal range. Although increased, ALT activity was not related to more severe course of SARS infection defined by high fever >39 °C, decreased blood oxygen saturation (SaO_2_) and immune functional disorder. Liver biopsy in four patients did not reveal any specific findings. All patients included in the study were treated with antibiotics which could have influenced ALT activity. Chronic hepatitis B was not associated with worse liver injury. The authors concluded that liver damage of patients with SARS usually occurs in the early stage of the disease characterized by decreased albumin levels and abnormal levels of ALT. The liver damage induced by SARS seems to be primary rather than secondary due to low SaO_2_ or hyperthermia. Hepatotoxic drugs may play a role in increasing the severity of liver damage or prolonging the time of liver function recovery.

The association between increased ALT that occurs at the very early stage of SARS and recognition that ACE2 is an entry receptor for SARS-CoV-1 suggests that the liver is a direct target of viral infection. Liver biopsies of patients with SARS have shown a significant increase in mitotic cells, with eosinophilic bodies and ballooning hepatocytes, suggesting that SARS-CoV-1 may induce apoptosis of liver cells and thus cause liver injury [13]. Other studies showed that SARS-CoV-specific protein 7a can induce apoptosis in cell lines of different organs (including the lung, kidney and liver) through the caspase-dependent pathway, further supporting the pathogenic role of SARS-CoV-1 on hepatocytes and cholangiocytes resulting in regeneration derangement and liver injury [82].

Duan et al. [83] found ALT activity to be elevated in almost 38% out of 154 SARS patients, with higher levels correlated with more severe disease course at the day of admission. ALT normalized in 75% out of those patients within two weeks. Serum albumin decreased in 29% of subjects. IL-1beta, IL-2, IL-4, IL-6, IL-8, IL-10, tumor necrosis factor (TNF)-alpha levels were upregulated in SARS patients within the first week of hospitalization and decreased by the fourth week. This increased cytokine expression was associated with higher ALT activity. Liver ultrasonography performed in 11 patients and histopathological examination of liver specimens in four patients did not show any characteristic features. The results suggest liver injury which occurs at the very early stage of infection to be part of a complex systemic inflammatory response syndrome (SIRS). Effective depletion of the cytokine storm may be beneficial to reduce liver impairment and improve SARS outcomes.

### 4.6. MERS-CoV and Liver Injury

MERS-CoV infection in patients is characterized by fever, cough and shortness of breath, respiratory failure in some cases and gastrointestinal syndromes affecting about 25%–30% of patients. MERS-CoV can cause severe infection requiring intensive care and mortality reaching 60%. Most MERS patients had upregulated ALT and AST activity and hyperbilirubinemia with average increase 2 × ULN, 3 × ULN and 1.5 × ULN, respectively. Hypoalbuminemia was another common finding in MERS patients at the day of diagnosis with frequent deterioration during the hospitalization. Concomitant infections and low albumin were independent risk factors for severe infection requiring ICU (OR 14.13, 95% CI 1.58–126.09; *p* = 0.018 and OR 6.31, 95% CI 1.24–31.90; *p* = 0.026, respectively) [52]. Similar observations were found in several other studies of MERS [52,84,85,86]. Similar to the observation in SARS patients, the pathological features of liver injury in MERS were mild portal tract and lobular lymphocytic infiltration, moderate steatosis and scattered calcifications [53]. Unlike SARS-CoV-2 and SARS-CoV-1, MERS-CoV required dipeptidyl peptidase-4 (DPP-4) as its functional receptor to enter cells and evoke infection in cells [87,88,89]. The lack of data makes it difficult to determine if the liver injury observed during MERS-CoV infection is the consequence of direct viral infection or is inflammation-mediated.

## 5. Conclusions

SARS-CoV-2, SARS-CoV-1 and MERS-CoV frequently affect the liver; however, numerous confounding factors could bias the interpretation of available data regarding the correlation between infection and hepatocyte damage. Pre-existing liver disease such as NAFLD and NASH or viral hepatitides were underreported in most the studied cohorts. Abnormal liver tests could be related to SARS, other pre-existing comorbidity treatment or other co-therapy. Liver injury which is diagnosed at the early stage of viral infection may result either from a direct insult of the virus or comprises part of the complex systemic inflammatory response syndrome associated with the infection. The mechanism of hepatic injury in SARS infections is still not elucidated, it is likely multifactorial and requires further prospective and properly designed studies. Medication used to treat COVID-19, SARS and MERS including interferon-α sprays, arbidol hydrochloride, lopinavir, ritonavir, antibiotics and glucocorticoids, may also account for hepatic dysfunction, but were underreported in the studied cohorts. More studies on the correlation between coronaviruses, especially SARS-CoV-2 and liver injury are necessary, including histopathology and molecular diagnostic assessments. An interaction between viral shedding of SARS-CoV-2 in stool has been reported [90], but it’s association with hepatotoxicity is yet to be determined.

## Figures and Tables

**Table 1:** Study and patient characteristics.

Reference/Year/Country	*n* Total/ *n* Males	Age (Mean/SD)	Mechanical Ventilation/Suppl Oxygen	Hospitalization/ICU Stay	Hospital Mortality/ICU Mortality	Comorbidities (Diabetes/Hypertension/CVD/Other)	Preexisting Liver Disease (*n*)/Diagnosis	Intervention
**SARS CoV-2**								
Chen et al. [16]/2020/China	99/67	55.5/13.1	17/75	99/nd	31/nd	nd/nd/nd/0	nd/nd	antibiotic, antiviral treatment
Chuan et al. [17]/2020/China	32/nd	nd/nd	nd/nd	32/nd	nd/nd	nd/nd/nd/5	nd/nd	nd
Guan et al. [2]/2020/China	1099/640	47/35.58	81/454	1029/55	15/nd	81/165/27/261	23/HBV	antibiotics (*n* = 637), oseltamivir (*n* = 393), antifungals (*n* = 31), systemic glucocorticoids (*n* = 204)
Wang et al. [12]/2020/China	138/75	56/26	32/106	138/36	6/nd	14/43/20/0	4/chronic liver disease	moxifloxacin (*n* = 89), ceftriaxone (*n* = 34), azithromycin (*n* = 25), glucocorticoids (*n* = 62)
Zhou et al. [18]/2020/China	191/nd	nd/nd	58/41	191/50	54/nd	36/58/15/22	nd/nd	antibiotics (*n* = 181), antivirals (*n* = 41), corticosteroids (*n* = 57), immunoglobulins (*n* = 46)
Zhang et al. [19]/2020/China	56/nd	nd/nd	nd/nd	56/nd	nd/nd	nd/nd/nd/0	2/nd	nd
Yang et al. [20]/2020/China	52/35	59.7/13.2	37/33	52/52	nd/32	9/nd/7/34	nd/nd	vasoconstrictive agents (*n* = 18), antivirals (*n* = 23), antibacterials (*n* = 49),glucocorticoids (*n* = 30), immunoglobulin (*n* = 28)
Xu et al. [21]/2020/China	62/35	41/20	1/nd	61/1	0/0	1/5/nd/3	7/nd	antivirals (*n* = 55), antibiotics (*n* = 28), systematic corticosteroid (*n* = 16)
Wu et al. [22]/2020/China	80/39	46.1/15.42	0/35	80/nd	0/0	nd/nd/25/12	1/nd	antibiotic treatment (*n* = 73), antivirals (*n* = 80), hormone therapy (*n* = 12), immunoglobulins (*n* = 16)
Shi et al. [23]/2020/China	81/42	49.5/11	nd/nd	81/nd	3/nd	10/12/8/0	7/liver cirrhosis, hepatitis	nd
Jin et al. [4]/2020/China	651/331	45.21/14.42	17/nd	nd/17	nd/nd	48/100/5/8	25/nd	antivirals (*n* = 546), antibiotics (*n* = 277), glucocorticoids (*n* = 74),
**SARS CoV-1**								
Chan et al. [24]/2004/China	118/55	33 */(20–18) ^#^	16/nd	nd/nd	9/nd	nd/nd/nd/16	12/HBV	lamivudine
Chan et al. [25]/2005/China	294/126	36 */(12–83) ^#^	33/nd	194/141	27/nd	5/12/6/18	30/HBV	cefotaxime, clarithromycin, oseltamivircorticosteroids, ribavirin, lamivudine
Chau et al. [13]/2004/China	3/0	34.7/8.2	nd/nd	3/nd	3/nd	nd/nd/nd/nd	nd/nd	ceftriaxone, clarithromycin, Kaletra, methylprednisolone or levofloxacin alone
Chen et al. [26]/2003/China	7/nd	nd/nd	nd/nd	nd/nd	nd/nd	nd/nd/nd/nd	nd/nd	nd
Cui et al. [27]/2004/China	182/103	nd/(11–86) ^#^	nd/nd	57/nd	nd/nd	nd/nd/nd/nd	nd/nd	antibiotics (*n* = 160), ribavirin (*n* = 137),methylprednisolone (*n* = 115)
Ding et al. [28]/2003/China	3/2	48/16.4	nd/nd	nd/nd	nd/nd	nd/nd/nd/nd	nd/nd	nd
Farcas et al. [29]/2005/Canada	21/9	68.8/15	nd/nd	nd/nd	nd/nd	6/9/3/16	nd/nd	nd
Guan et al. [30]/2004/China	110/nd	nd/nd	nd/nd	nd/nd	8/nd	nd/nd/nd/nd	nd/nd	nd
Han et al. [31]/2003/China	69/29	nd/nd	nd/nd	nd/nd	nd/nd	nd/nd	nd/nd	nd
Hsiao et al. [32]/2004/Taiwan	346/nd	nd/nd	nd/nd	nd/nd	73/nd	nd/nd/nd/nd	nd/nd	nd
Kumar et al. [33]/2003/Canada	1/1	74/0	nd	1/1	1/1	nd/nd/nd/nd	nd/nd	cyclosporin, prednisone, insulin, trimethoprim/sulfamethoxazole prophylaxis
Lang et al. [34]/2003/China	3/nd	nd/nd	nd/nd	nd/nd	nd/nd	nd/nd/nd/nd	nd/nd	nd
Liu et al. [35]/2003/China	106/56	36/10	nd/nd	nd/nd	nd/nd	nd/nd/nd/nd	nd/nd	steroids, antibiotics, antiviral drugs
Luo et al. [36]/2003/Germany	1/1	54/nd	1/nd	1/1	0/0	nd/nd/nd/nd	nd/nd	ribavirin
Zhao et al. [37]/2004/China	106/nd	nd/nd	nd/nd	nd/nd	nd/nd	nd/nd/nd/nd	nd/nd	nd
Yin et al. [38]/2004/China.	148	nd/nd	nd/nd	nd/nd	nd/nd	nd/nd/nd/nd	nd/nd	nd
Yang et al. [39]/2005/China	168/72	42.8/18.6	nd/nd	nd/nd	nd/nd	nd/nd/nd/nd	17/HBV	quinolones, macrolides, floxacin, tetracycline, roxithromycin, ciprofloxacin
Wu et al. [40]/2004/ Taiwan	52/20	45/20	nd/nd	nd/21	16/nd	nd/nd/nd/nd	8/HBV	nd
Wong et al. [41]/2003/China	54/24	37.9/13	nd/nd	nd/nd	nd/nd	nd/nd/nd/nd	nd/nd	Corticosteroids and oral (or iv) ribavirin, cefipime, oral clarithromycin, azithromycin
Tong et al. [42]/2003/China	114/nd	nd/nd	nd/nd	nd/nd	nd/nd	nd/nd/nd/nd	nd/nd	nd
Shi et al. [43]/2005/China	7/6	40.43/13.95	nd/nd	nd/nd	nd/nd	nd/nd/nd/nd	nd/nd	nd
Peiris et al. [45]/2003/China	50/22	42.99/12.58	19/nd	/nd19	1/nd	nd/nd/nd/nd	nd/nd	Oral levofloxacin (*n* = 9), amoxicillin-clavulanate (given intravenously *n* = 40), oseltamivir orally (*n* = 4), intravenous ceftriaxone, Azithromycin, oral amantadine (*n* = 1), intravenous ribavirin, steroid (*n* = 49)
Meng et al. [44]/2003/China	41/8	nd/nd	27/11	nd/nd	1/nd	nd/nd/nd/nd	nd/nd	Steroids
**MERS CoV**								
Al Tawfiq et al. [46]/2017/USA	16/nd	nd/nd	nd/nd	15/nd	nd/nd	nd/nd/nd/nd	nd	nd
Alsaad et al. [8]/2018/Saudi Arabia	1/1	33/nd	1/nd	1/1	1/1	nd/nd/nd/1	nd	Chemotherapy, methotrexate, antibiotics ifosfamide, etoposide, L-asparginase, prednisolone
Halim et al. [47]/2016/Egypt	32/20	43.99/13.03	23/nd	32/32	14/14	nd/nd/nd/31	nd	nd
Ling et al. [48]/2015/China	1/nd	43/nd	1/nd	1/nd	nd/nd	nd/nd/nd/nd	nd	Ribavirin, ceftriaxone, meropenem
Kapoor et al. [49]/2014/USA	1/1	65/nd	0/nd	1/0	0/0	nd/1/1/1	nd	vancomycin, piperacillin/, ceftriaxone tazobactam, levofloxacin, linezolid, furosemide
Yousefi et al. [50]/2017/Iran	5/1	49.6/10.52	nd/nd	4/nd	3/3	nd/1/nd/1	nd	PT1: azithromycin, ceftriaxone, meropenem, vancomycin, oseltamivir; PT2: levofloxacin, ceftriaxone, azithromycin, oseltamivir; PT3: no drugs, P4: no data (pt. died in ICU), P5: meropenem and vancomycin, oseltamivir
Sherbini et al. [51]/2017/Saudi Arabia	29/20	45.49/12.22	9/nd	nd/nd	10/nd	9/nd/nd/8	nd	Meropenem (*n* = 20), linezolid (*n* = 17), levofloxacin (*n* = 15), piperacillin (*n* = 15), ribavirin (*n* = 10), azithromycin (*n* = 19), interferon (*n* = 19), steroids (*n* = 29)
Saad et al. [52]/2014/Saudi Arabia	70/46	61 */(1–90)Z	49/nd	nd/49	42/nd	nd/nd/nd/nd	nd	nd
Ng et al. [53]/2014/United Arab Emirates	1/1	45	1/nd	nd/nd	nd/nd	nd/nd/nd/nd	nd	Prednisolone, paracetamol, levofloxacin, oseltamivir, ceftriaxone, azithromycin, hydrocortisone intravenously

^#^ min and max; * median; Z, interquartile range (IQR); nd, no data;. HBV, hepatitis B virus; SD, standard deviation; ICU, intensive care unit; CVD, cardiovascular disease; SARS CoV-1, severe acute respiratory syndrome coronavirus 1; SARC-CoV-2, severe acute respiratory syndrome coronavirus 2; MERS-CoV, Middle East respiratory syndrome coronavirus.

**Table 2:** Liver-related outcomes in presented studies.

Reference	*n* Total	AST (Mean) ALT (Mean) LDH (Mean)	Abnormal AST (*n*) Abnormal ALT (*n*) Abnormal LDH (*n*)	Bilirubin (Mean) ALP (Mean) Creatinine (Mean)	Abnormal Bilirubin (*n*) Abnormal ALP (*n*) Abnormal Creatinine (*n*)	Total Protein (Mean) Albumin (Mean)	Abnormal Tot. Protein (*n*) Abnormal Albumin (*n*)	Prothrombin Time (Mean) INR (Mean)	Abnormal Prothrombin Time (*n*) Abnormal INR (*n*)	Abnormal Liver Function (*n*/*n* Total)
**SARS CoV-2**										
Chen et al. [16]/2020/China	99	nd	AST: 35 ALT: 28 LDH: 75	nd	BIL: 18 CR: 24	nd	nd	nd	nd	nd
Chuan et al. [17]/2020/China	32	AST (U/L): 24.75 ALT (U/L): 26.98	nd	BIL (mmol/L): 16.4	nd	ALB (g/L): 39	nd	nd	nd	nd
[2]/2020/China	1099	nd	AST: 168 ALT: 158 LDH: 277	nd	BIL: 76 CR: 12	nd	nd	nd	nd	nd
Wang et al. [12]/2020/China	138	AST (U/L):31 * ALT (U/L): 24 * LDH (U/L): 261 *	nd	BIL (mmol/L): 9.8 * CR (µmol/L): 72 *	nd	nd	nd	PT (s): 13 *	nd	nd
Zhou et al. [18]/2020/China	191	ALT (U/L): 30 * LDH (U/L): 300 *	ALT: 59 LDH: 123	nd	CR: 8	ALB (g/L): 32.3 *	nd	PT (s): 11.6 *	PT: 182	nd
Zhang et al. [19]/2020/China	56	nd	nd	nd	ALP: 1	nd	nd	nd	nd	16/56
Yang et al. [20]/2020/China	52	nd	nd	BIL (µmol/L): 17.04 CR (µmol/L): 79	nd	nd	nd	PT (s): 12.3	nd	nd
Xu et al. [21]/2020/China	62	AST(U/L): 26 * ALT (U/L):22* LDH (U/L): 205 *	AST: 10 LDH: 17	CR (µmol/L): 72	CR: 3	nd	nd	nd	nd	nd
Wu et al. [22]/2020/China	80	AST (U/L): 30 * ALT (U/L): 24 * LDH (U/L): 226 *	AST: 3 ALT: 3 LDH: 17	BIL (µmol/L): 6.6 CR (µmol/L): 78	BIL: 1 CR: 2	ALB (g/L): 38.3 *	ALB: 2	PT (s): 10.8	nd	nd
Shi et al. [23]/2020/China	81	AST (U/L): 40.8 ALT (U/L): 46.2	AST: 43	BIL (µmol/L): 11.9 CR (µmol/L): 75.4	nd	ALB (g/L): 32.9	nd	PT (s): 10.7	nd	nd
Jin et al. [4]/2020/China	651	AST (U/L): 29.35 (g)/24.2 (ng) * ALT (U/L): 25(g)/21.5 (ng) LDH (U/L): 229(g)/210 (ng)	nd	BIL (µmol/L): 10 (g)/9.6 (ng) CR (µmol/L):66.0 (g)/66.0 (ng)	nd	ALB (g/L): 40.13 (g)/41.5 (ng)	nd	nd	INR: 1.03 (g)/1.02 (ng)	nd
**SARS CoV-1**										
Chan et al. [24]/2004/China	118	ALT (U/L): 25.5	ALT: 25	CR (µmol/L): 85	nd	nd	nd	PT (s): 11.2 *	nd	nd
Chan et al. [25]/2005/China	294	nd	ALT: 52	nd	ALP: 40	nd	nd	nd	nd	nd
Chau et al. [13]/2004/China	3	ALT (U/L): 165X	nd	BIL (µmol/L): 7.3X	nd	ALB (g/L): 32Y	nd	nd	nd	nd
Chen et al. [26]/2003/China	7	nd	nd	nd	nd	nd	nd	nd	nd	nd
Cui et al. [27]/2004/China	182	nd	ALT: 89 AST: 89 LDH: 76	nd	nd	nd	nd	nd	nd	nd
Ding et al. [28]/2003/China	3	nd	nd	nd	nd	nd	nd	nd	nd	nd
Farcas et al. [29]/2005/Canada	21	nd	nd	nd	nd	nd	nd	nd	nd	nd
Guan et al. [30]/2004/China	110	ALT (U/L): 91.61X AST (U/L): 78.68X LDH (U/L): 429.69X	nd	BIL (µmol/L): 11.67X	ALP: 0	ALB (g/L): 34.4	nd	nd	nd	nd
Han et al. [31]/2003/China	69	nd	nd	nd	nd	nd	nd	nd	nd	37/nd
Hsiao et al. [32]/2004/Taiwan	346	nd	nd	nd	nd	nd	nd	nd	nd	nd
Kumar et al. [33]/2003/Canada	1	AST (U/L):51	ALT: 1	nd	BIL: 1	nd	nd	nd	nd	nd
Lang et al. [34]/2003/China	3	nd	nd	nd	nd	nd	nd	nd	nd	nd
Liu et al. [35]/2003/China	106	nd	ALT: 8	nd	nd	nd	nd	nd	nd	nd
Luo et al. [36]/2003/Germany	1	ALT (U/L): 425.5X AST (U/L): 319X	LDH: 0	nd	CR: 0	nd	nd	nd	nd	nd
Zhao et al. [37]/2004/China	106	nd	ALT: 106 AST: 68	nd	nd	nd	TP: 0 ALB: 122	nd	nd	nd
Yin et al. [38]/2004/China	148	nd	nd	nd	nd	nd	nd	nd	nd	148
Yang et al. [39]/2005/China	168	ALT (U/L): 111.32X AST (U/L): 48.95X	ALT:118	BIL: (µmol/L): 10.41X	nd	ALB (mg/L): 34.26Y	nd	nd	nd	nd
Wu et al. [40]/2004/ Taiwan	52	ALT (U/L): 86.19X AST (U/L): 69.05 X	ALT: 28 AST: 28	nd	nd	nd	nd	nd	nd	nd
Wong et al. [41]/2003/China	54	ALT (U/L): 95.7X AST (U/L): 62.8X	ALT:41X	BIL: (µmol/L): 11.1X ALP (U/L): 72.6X	nd	ALB (g/L): 33.2Y	nd	nd	61	nd
Tong et al. [42]/2003/China	114	nd	nd	nd	nd	nd	nd	nd	nd	84/nd
Shi et al. [43]/2005/China	7	nd	AST: 4	nd	nd	nd	nd	nd	nd	nd
Peiris et al. [45]/2003/China	50	ALT (U/L): 63 *	ALT: 17	nd	nd	ALB (g/L): 37	ALB: 34	nd	nd	17/nd
Meng et al. [44]/2003/China	41	nd	nd	nd	nd	nd	nd	nd	nd	27/nd
**MERS CoV**										
Al Tawfiq et al. [46]/2017/USA	16	AST (U/L): 661X ALT (U/L): 476X LDH (U/L): 1825.8X	nd	BIL (µmol/L): 21X ALP (U/L): 257.3X CR (mg/dL): 3.8X	nd	nd	nd	nd	nd	nd
Alsaad et al. [8]/2018/Saudi Arabia	1	nd	nd	nd	nd	nd	nd	nd	nd	0/1
Halim et al. [47]/2016/Egypt	32	nd	nd	nd	nd	nd	nd	nd	nd	nd
Ling et al. [48]/2015/China	1	nd	nd	nd	nd	nd	nd	nd	nd	nd
Kapoor et al. [49]/2014/USA	1	AST (U/L): 95 ALT (U/L): 80	nd	BIL (mg/dL): 1 ALP (U/L) 270 CR (mg/dL): 0.75	nd	nd	nd	nd	nd	nd
Yousefi et al. [50]/2017/Iran	5	AST (U/L): 60.7 ALT (U/L): 35.75	AST: 3 ALT: 2	CR (mg/dL): 0.77	nd	nd	nd	PT: 13.65 INR: 1.1	PT: 2 INR: 1	nd
Sherbini et al. [51]/2017/Saudi Arabia	29	AST (U/L): 86.3 ALT (U/L): 98.4	nd	BIL (µmol/L): 16.64 CR (µmol/L): 225	nd	nd	nd	nd	nd	nd
Saad et al. [52]/2014/Saudi Arabia	70	AST (U/L): 112 * XALT (U/L): 54 *X	nd	BIL (µmol/L): 17 * XALP (U/L): 145 * XCR (µmol/L): 251.5 *X	nd	ALB (mg/dL): 21 *Y	nd	nd	nd	22/70
Ng et al. [53]/2014/United Arab Emirates	1	AST (U/L): 51 ALT (U/L): 28	nd	CR (mg/dL): 0.9	nd	nd	nd	PT: 12 INR: 1.1	nd	nd

* median; X, highest measurement during the study; Y, lowest measurement during the study; nd, not determined; AST, aspartate aminotransferase; ALT, alanine aminotrasfesrase; LDH, lactate dehydrogenase; ALP, alkaline phosphatase; INR, international normalized ratio; BIL, bilirubin; ALB, albumin; CR, creatinine; PT, prothrombin time.

**Table 3:** Histopathological findings within the livers of patients infected with coronaviruses.

Reference	Post-Mortem Study (Y/N)	Type of Coronavirus	Histopathology Cases (in Words)
[24]	N	SARS-CoV-1	• no acute changes, no necrosis
[13]	N	SARS-CoV-1	• mild lobular activities with occasional acidophilic bodies and prominent Kupffer cell • smildly inflamed portal tracts with lymphocytic infiltration
[26]	Y	SARS-CoV-1	• massive necrosis (1 case) • nodular cirrhosis (1 case)
[28]	Y	SARS-CoV-1	• dissociation of hepatocyte cords, together with fatty degeneration and focal necrosis (1 case) • massive central necrosis of hepatocytes (2 cases) • the vascular walls with edema and infiltration of monocytes and lymphocytes
[29]	Y	SARS-CoV-1	• minor inflammatory changes observed in the liver on microscopic examination
[30]	N	SARS-CoV-1	• non-specific inflammation in the liver in biopsy • non-specific hepatitis in postmortem biopsy
[32]	N	SARS-CoV-1	• no specific pathological change in the gastrointestinal tract
[34]	Y	SARS-CoV-1	• hydropic degeneration • fatty degeneration • interstitial cell proliferation
[39]	N	SARS-CoV-1	• hydropic degeneration • steatosis • focal necrosis (*n* = 4)
[43]	Y	SARS-CoV-1	• mild fatty-acid degeneration • mild congestion • central lobular necrosis
[8]	Y	MERS-CoV	• mild chronic lymphocytic portal inflammation • reactive parenchyma with mild cellular hydropic degeneration • rare multinucleated hepatocytes and mild disarray of the hepatic plates • mild sinusoidal lymphocytosis and small necroinflammatory foci in the hepatic lobules • congestion, hemorrhage and focal perivenular loss of hepatocytes • macrovesicular perivenular steatotic change, sinusoidal congestion, hemorrhage and focal perivenular loss of hepatocytes
[53]	Y	MERS-CoV	• moderate steatosis • scattered calcifications • mild portal tract and lobular lymphocytic inflammation

SARS-CoV-1, severe acute respiratory syndrome coronavirus 1; MERS-CoV, Middle East respiratory syndrome coronavirus; N, no; Y, yes.

## Supplementary Materials

The following are available online at https://www.mdpi.com/2077-0383/9/5/1420/s1, Table S1: Risk of bias assessment.
